# Primary care characteristics and their association with health screening in a low-socioeconomic status public rental-flat population in Singapore- a mixed methods study

**DOI:** 10.1186/s12875-016-0411-5

**Published:** 2016-02-06

**Authors:** Liang En Wee, Wen Qi Cher, David Sin, Zong Chen Li, Gerald Choon-Huat Koh

**Affiliations:** Singhealth Internal Medicine, Singapore General Hospital, Singapore, Singapore; Yong Loo Lin School of Medicine, National University of Singapore, National University Health System, Singapore, Singapore; Saw Swee Hock School of Public Health, National University of Singapore, National University Health System, #10-03-G, Tahir Foundation Building, Block MD1, 12 Science Drive 2, Singapore, Singapore

**Keywords:** Primary care, Low-income, Health screening, Chronic disease

## Abstract

**Background:**

In Singapore, subsidized primary care is provided by centralized polyclinics; since 2000, policies have allowed lower-income Singaporeans to utilize subsidies at private general-practitioner (GP) clinics. We sought to determine whether proximity to primary care, subsidised primary care, or having regular primary care associated with health screening participation in a low socioeconomic-status public rental-flat community in Singapore.

**Methods:**

From 2009–2014, residents in five public rental-flat enclaves (*N* = 936) and neighboring owner-occupied precincts (*N* = 1060) were assessed for participation in cardiovascular and cancer screening. We then evaluated whether proximity to primary care, subsidised primary care, or having regular primary care associated with improved adherence to health screening. We also investigated attitudes to health screening using qualitative methodology.

**Results:**

In the rental flat population, for cardiovascular screening, regular primary care was independently associated with regular diabetes screening (adjusted odds ratio, aOR = 1.59, CI = 1.12–2.26, *p* = 0.009) and hyperlipidemia screening (aOR = 1.82, CI = 1.10–3.04, *p* = 0.023). In the owner-occupied flats, regular primary care was independently associated with regular hypertension screening (aOR = 9.34 (1.82–47.85, *p* = 0.007), while subsidized primary care was associated with regular diabetes screening (aOR = 2.94, CI = 1.04–8.31, *p* = 0.042). For cancer screening, in the rental flat population, proximity to primary care was associated with less participation in regular colorectal cancer screening (aOR = 0.42, CI = 0.17–0.99, *p* = 0.049) and breast cancer screening (aOR = 0.29, CI = 0.10–0.84, *p* = 0.023). In the owner-occupied flat population, for gynecological cancer screening, usage of subsidized primary care and proximity to primary care was associated with higher rates of breast cancer and cervical cancer screening; however, being on regular primary care followup was associated with lower rates of mammography (aOR = 0.10, CI = 0.01–0.75, *p* = 0.025). On qualitative analysis, patients were discouraged from screening by distrust in the doctor-patient relationship; for cancer screening in particular, patients were discouraged by potential embarrassment.

**Conclusions:**

Regular primary care was independently associated with regular participation in cardiovascular screening in both low-SES and higher-SES communities. However, for cancer screening, in the low-SES community, proximity to primary care was associated with less participation in regular screening, while in the higher-SES community, regular primary care was associated with lower screening participation; possibly due to embarrassment regarding screening modalities.

**Electronic supplementary material:**

The online version of this article (doi:10.1186/s12875-016-0411-5) contains supplementary material, which is available to authorized users.

## Background

Access to healthcare can be subdivided into socioeconomic aspects, such as cost, attitudes, and belief; as well as geographic access, including physical distance, travel times, and convenience. A distinction can be drawn between the physical characteristics of primary care (affordability, in terms of cost; availability, in terms of proximity, accessibility and opening hours) and the interaction between physician and patient, which is influenced by factors such as approachability, acceptability and appropriateness [1]. In Western urbanized societies, access to primary care is high, with greater proportions of primary care providers in more deprived areas [2, 3]. Accessibility of primary care and preventive health services is related to geographic distance, especially in rural areas [4, 5]. In particular, those living in deprived areas are more dependent on health services within their neighborhoods, because of reduced mobility and resources [6]. However, these studies are mainly focused on Western urbanized populations; within the literature, few studies have examined the relationship between primary care characteristics and primary care utilisation in Asian societies. As the type of practice setting can influence doctors’ empathy and patients’ enablement [7] and the patient-physician interaction is important in encouraging screening participation in low-income populations [8], we were interested in determining whether the physical characteristics of primary care (affordability and availability) and the doctor-patient relationship (approachability) influenced screening participation in a low-income Asian community,

Singapore is an example of an urbanized, multi-ethnic Asian society. Primary care is provided by government-run primary care clinics called polyclinics and private general medical practitioner (GP) clinics [9]. Polyclinics provide about 20 % of primary health care while private GPs provide the remaining 80 %. Although private GPs enjoy certain advantages, such as greater continuity of care, shorter wait time lesser patient load and greater spatial accessibility [10–12], the majority still seek treatment mainly from polyclinics. Reasons include: geographical convenience, subsidies, and comprehensive facilities including onsite laboratory and imaging services [11, 13]. A more detailed comparison of private GPs and government-run polyclinics is found in Table 1. Access to primary care amongst those staying in low socioeconomic status (SES) areas is more limited. The main area-level indicator of SES in Singapore is home ownership. The majority (≥85 %) stay in owner-occupied public housing and due to government subsidies, home ownership is high (90.3 %) [14]. Public rental flats provide heavily subsidized rentals for the needy (3.7 %) of the population, 88 % of whom earn less than S$670/month [15]. These public rental flat neighborhoods are scattered across Singapore, forming low-SES enclaves immediately adjacent to neighboring precincts of owner-occupied public housing. Our previous studies in these neighborhoods showed that only a small minority preferred to approach family physicians for primary care- the large majority turned to alternative medicine (eg. traditional Chinese medicine), family, or self-reliance [16]. In addition, access to preventive services, like cancer and cardiovascular screening, is poorer in these low-SES areas [17, 18]; and management of chronic diseases like hypertension is less optimal [19]. Allowing lower-income Singaporeans to receive subsidized care at private GPs provides the potential of greater choice, convenience and continuity of care; however, the effectiveness of this has not been evaluated. While our previous local studies identified various barriers to screening access (cost, knowledge, attitudes, convenience, the doctor-patient relationship), we did not determine which of these barriers were most significant in the low-SES population.

**Table 1:** Comparison of private general practitioner clinics and government-run polyclinics in Singapore

Primary care characteristics	Private general practitioner (GP) clinic	Government-run polyclinic (Primary care clinic)
Service provision:		
Number of clinics [9]	~2000 clinics nation-wide	18 government polyclinics
Percentage of primary healthcare visits (acute and chronic conditions) [10]	80 % of primary healthcare visits	20 % of primary healthcare visits
Percentage of primary healthcare visits for chronic disease [10]	55 % of primary healthcare visits for chronic disease	45 % of primary healthcare visits for chronic disease
Services provided [9]	Comprising solo, small group or large health care group practices. Usually do not possess onsite investigative and laboratory services. Community Health Centres provide off-site ancillary support services to GPs without full facilities.	Complete range of medical care for both acute and chronic medical conditions, including health screening, outpatient medical care, x-ray and laboratory services
Availability of cancer and cardiovascular screening [32]	Blood pressure screening and fasting blood tests for diabetes/dyslipidemia are widely available.	All screening tests generally available.
		Not all polyclinics have mammography facilities; sometimes referred to more central polyclinics.
	Mammograms are usually by referral to off-site facilities.	
	Provision of pap smear/fecal occult blood test varies.	
Characteristics of primary care:		
Availability of subsidised care [9, 33]	Usually unsubsidised.	Singapore citizens above 65 receive up to 75 % concessions in consultation and treatment fees, while all other Singapore citizens are given a 50 % concession
	However, under the Community Health Assist Scheme, those eligible get 80–$120 subsidy per visit for chronic diseases; free screening tests; and $18.50 subsidy per visit for doctor’s consultation for health screening.	
Continuity of care [9]	Greater continuity of care as usually one main family physician at private clinics	Patients are usually assigned any doctor from a common group of medical officers and family physicians.
		They may also choose to see the doctors from the Family Physician Clinic in the polyclinic which ensures them care continuity from the same doctor, but at a higher rate.
Patient load [10]	Around 30 patients/day for each doctor	Around 58 patients/day for each doctor
Wait time [34]	Wait time for registration and consultation is usually around 5–10 min	Wait time at registration can range from 13 to 69 min; wait time for consultation can range from 43 to 112 min
24 h coverage [9]	Some GPs may offer 24 h coverage	Do not offer 24 h coverage. Patients may visit 24 h A&E (accident and emergency) departments when necessary.
Geographical proximity	Most public housing estates have at least one GP clinic within walking distance.	Patients usually have to travel about 3 km to the nearest polyclinic. There may be shuttle services provided from nearby transport nodes (eg. bus interchanges/train stations).
	Densities of GP clinics may be lower in less mature estates.	
Usage of traditional/alternative medicine [16]	Generally not provided.	Generally not provided.
	Traditional Chinese medicine is provided at separately licensed traditional Chinese medicine practitioners; not subsidised by the public healthcare system.	Traditional Chinese medicine is provided at separately licensed traditional Chinese medicine practitioners; not subsidised by the public healthcare system.
Communication barriers [16]	Usually less difficulties with communication as GPs are based in the neighbourhood and thus may have a better knowledge of their community.	As the polyclinics may be located at a distance from patients’ homes, the doctors at the polyclinic may not know so much detail about patients’ communities.
		In addition, some of the doctors at the polyclinic may be foreign-trained and have some communication difficulties with the local language.

We therefore sought to determine whether greater availability, in the form of physical proximity to primary care (eg. GP or public polyclinic),greater affordability, from being a recipient of subsidized care, and greater approachability and acceptability, in the form of having a closer doctor-patient relationship via regular followup with a primary care doctor, were associated with more regular participation in cancer and cardiovascular screening, in low socioeconomic-status public rental-flat communities in Singapore. In addition, to better understand the attitudes and barriers of residents in these low-SES areas towards participation in health screening, we conducted a qualitative study with residents of these neighborhoods. We hope that these results will aid in addressing the issue of access to primary care and health services in similar resource-poor settings, particularly in urbanized Asian societies.

## Methods

From 2009 to 2014, residents in five public rental-flat enclaves (*N* = 936); as well as residents in neighbouring owner-occupied precincts (*N* = 1060) were assessed for participation in cardiovascular and cancer screening. We then evaluated whether various primary care characteristics were associated with improved health screening adherence in the rental population, comparing against residents staying in adjacent owner-occupied public housing.

### Study population

The study population consisted of all Singaporean citizens/permanent residents aged ≥ 40 years, living in five integrated public housing precincts in Singapore, recruited between 2009 and 2014. In Singapore, due to high urban density, blocks of public rental housing (lower area-SES) and public owner-occupied housing (higher area-SES) occupy the same geographical space, forming integrated public housing precincts. Site A, in Western Singapore, contained 3 blocks of public rental flats and 4 blocks of owner-occupied housing; Site B, in Eastern Singapore, contained 4 blocks of public rental flats and 5 blocks of owner-occupied housing; Site C, in Eastern Singapore, contained 3 blocks of public rental flats and 5 blocks of owner-occupied housing; Site D, in Eastern Singapore, contained 2 blocks of public rental flats and 1 block of owner-occupied housing; while Site E, in Central Singapore, contained 2 blocks of public rental flats and 1 block of owner-occupied housing. Site A and C fall into the category of middle-aged estates (developed after 1980), while Site B, D and E fall into the category of mature estates (developed before 1980). These sites were chosen in order to give a good geographical spread of sites, as the bulk of public rental flats are scattered across the western, eastern and central housing estates of Singapore, with the majority in middle-aged/mature estates.

### Baseline information

At baseline, information such as sociodemographic data/medical history was collected during door-to-door visits via interviewer-administered standardized questionnaires in English, Chinese and Malay. Residents were asked for their full self-reported medical history and assessed if they were adherent to regular screening for cardiovascular disease (hypertension, diabetes, hyperlipidemia) and cancer (colorectal, cervical and breast cancer). Interviewers were medical students who underwent standardized training prior to study commencement. Ethics approval was obtained from the NUS Institutional Review Board, informed consent was sought, and participation was voluntary.

### Definitions

#### Primary care characteristics

We looked at the association between regular cancer/cardiovascular screening participation and the following aspects of primary care: 1) proximity to primary care (either a private GP, or a public polyclinic); 2) Receiving subsidised primary care, via the Community Health Assist (CHAS) programme; 3) Having regular follow-up with a primary care physician, instead of ad-hoc visits to primary care.

#### Proximity to primary care

In our communities, general private GP clinics were much nearer (within walking distance), compared to most of the polyclinics for which usage of public transport was necessary for access. Thus, we used walking distance to the nearest private GP clinic as a marker of proximity to the private GP clinic, and road distance to the nearest public polyclinic as a marker of proximity to public primary care services. These distances were computed using the postal codes of residents’ blocks, postal codes of the various private GP clinics [20] as well as postal codes of the public polyclinic in the vicinity. ArcGIS was used to calculate these distances [21]. In the rental flat population, the mean walking distance to a private GP clinic was 159 m, while the mean road distance to polyclinic was 3390 m. In the non-rental flat population (owner-occupied housing), the mean walking distance to a private GP clinic was 264 m, while the mean road distance to polyclinic was 3906 m. Thus, we defined proximity to primary care as staying either <150 m from a private GP clinic, in terms of walking distance; or staying <3.0 km from the public polyclinic, in terms of road distance.

#### Usage of subsidised primary care

We defined usage of subsidised primary care as answering “yes” to the question, “Do you have a Community Health Assist (CHAS) card?” In 2000, the Singapore government rolled out a scheme that allows lower-income Singaporeans to receive subsidies for medical treatment at GPs near their homes, now known as the Community Health Assist Scheme (CHAS) [22]. Those on public assistance and those with a monthly household income per person of < S$1800 (compared with the median Singaporean monthly household income per person of S$2380) [23] are eligible for participation in CHAS. Of note, the CHAS programme is an opt-in scheme. This means that patients must first register for the programme and obtain a card certifying that they are a member of the scheme, before they can be eligible for subsidies (even if they meet the income criteria).

#### Regular primary care

Patients were asked the question, “Are you on regular follow-up with a Western-trained doctor?” If they answered “yes” to the question, they were further asked to indicate where they were following up with: at the GP clinic, the polyclinic, free clinic, or the hospital. We defined receiving regular primary care as answering “yes” to the question, “Are you on regular follow-up with a Western-trained doctor”; and indicating that the doctor they were seeing was based at the GP clinic or the polyclinic. We excluded hospital-based doctors, as the majority of these would be specialists in other medical and surgical disciplines, not family medicine practitioners. We also excluded medical staff at free clinics. In Singapore, some voluntary welfare organisations set up free clinics to offer rudimentary free medical services to the low-income. However, given the limited scope of these services, the rudimentary nature, and the lack of continuity of care, we excluded these services from our definition of “regular primary care”.

#### Regular screening participation

Regular screening for cardiovascular disease (hypertension, diabetes, hyperlipidemia) and cancer (colorectal, cervical and breast cancer) was defined as adhering to the screening frequencies recommended by the local Ministry of Health [24].

#### Statistical analysis

Descriptive statistics were computed for the study population. We used Chi-square analysis to examine associations between sociodemographic factors, area-level SES (rental vs. owner-occupied), individual-level SES (education, employment) and usage of subsidised primary care, proximity to primary care and regular primary care. Subsequently, we identified factors independently associated with health screening participation using multivariate logistic regression, controlling for clustering at the block level. The criterion for initial entry of variables into multivariate models was *p* < 0.2 on univariate analysis. All statistical analysis was performed using SPSS (Version 17.0, USA) and STATA (Version 13.0, USA) and statistical significance was set at *p* < 0.05.

##### Study population (qualitative)

In order to better understand how attitudes and primary care characteristics shaped the willingness of residents in a low-SES neighbourhood to participate in health screening, we further conducted a qualitative study on this population. Patients were recruited via purposive sampling techniques from the quantitative study population, amongst those staying in public rental flats, and who did not participate in screening at baseline. Respondents were chosen to ensure roughly similar proportions of gender and ethnicities compared to the population-at-large. Participants were recruited via letters of invitation and were reimbursed S$10.

##### Conduct of interview sessions for qualitative study

Individual interviews (approximately an hour each) were carried out in residents’ homes. Interviewers were four medical students with extensive previous engagement (at least ≥1 year) in community outreach initiatives providing medical care to these needy communities. These interviewers underwent training by senior members of the study team (WLE, GCHK) prior to study commencement via a week-long workshop on qualitative/quantitative research skills. In addition, senior investigators (the first and last authors) demonstrated techniques of qualitative interviewing through active role-playing sessions, and in the initial interviews, supervised the medical students. Interviewers were matched to interviewees by race and language/dialect; the interview was conducted in the interviewee’s first language/dialect and audio-recordings were translated to English before qualitative content analysis. Interviewers used a standardized interview guide comprising a series of open-ended questions (Additional file 1: Table S1) to elicit interviewees’ perceptions about cardiovascular disease and cancer screening.

##### Qualitative content analysis

For the initial interview transcripts, investigators identified and highlighted every codable “unit of text” in the transcripts that represented a singular idea. Each unit of text was then reviewed and a list of themes representing distinct barriers/enablers to screening was created. All investigators then met to produce a master list comprising all unique themes identified. All accumulated transcripts were then recoded using the master list. The team met regularly, allowing addition of new themes to the master list as they arose. Additional residents were interviewed until saturation was reached. The final master list was then used by two senior investigators (WLE, GCHK) to independently review all transcripts and recode accordingly; finally meeting to compare recoded transcripts and resolve divergences.

## Results

### General population characteristics

Participation rates were 72.0 % (936/1300) for the rental flat communities and 58.9 % (1060/1800) for the owner-occupied communities, respectively. Of those staying in rental flats, a greater proportion were utilizing subsidized primary care in the form of the CHAS scheme (52.5 % vs. 20.5 %, OR = 4.29, CI = 3.52–5.22, *p* < 0.001) (Table 2). Staying in a public rental flat community was associated with greater proximity to primary care (94.7 % vs. 61.5 %, OR = 11.01, CI = 8.13–15.13, *p* < 0.001). However, despite having higher numbers on the CHAS scheme and greater spatial accessibility to primary care (geographical proximity), lesser proportions of residents in the rental flat community had regular followup with a primary care physician, compared with their counterparts staying in owner-occupied housing (52.2 % vs. 81.9 %, OR = 0.24, CI = 0.20–0.30, *p* < 0.001). Screening rates for both cardiovascular and cancer screening were generally lower in the public rental flat community, compared against both their counterparts staying in owner-occupied housing, and national statistics. For cardiovascular screening, in the rental flat community 44,3 % (255/575) were going for regular hypertension screening, 44.6 % (332/744) for regular diabetes screening and 35.0 % (224/640) for regular hyperlipidemia screening; this compared against national averages of 70.8 % for hypertension screening, 63.5 % for diabetes screening and 61.2 % for hyperlipidemia screening, respectively [25]. For cancer screening, in the rental flat community 8.3 % (60/722) were going for regular fecal occult blood testing; 18.0 % (60/334) were going for regular pap smears, and 13.3 % (69/517) were going for regular mammograms; this compared against national averages of ~40 % for pap smears and mammograms, and ~10 % for fecal occult blood testing [25].

**Table 2:** Characteristics of primary care and sociodemographic factors in 5 integrated public housing estates in Singapore from 2009 to 2014 (*N* = 1996)

	Owner-occupied blocks (higher-SES), *N* (%)	Rental flat blocks (low-SES), *N* (%)	OR (95 % CI)	*p*-value
	(*N* = 1060)	(*N* = 936)		
Site				
Middle-aged housing estate	75.2 (797/1060)	69.3 (649/936)	1.00	0.004
Mature housing estate	24.8 (263/1060)	30.7 (287/936)	1.34 (1.10–1.63)	
Primary care characteristics				
On subsidized primary care (CHAS scheme)				
Not on CHAS scheme	79.5 (843/1060)	47.5 (445/936)	1.00	<0.001
On CHAS scheme	20.5 (217/1060)	52.5 (491/936)	4.29 (3.52–5.22)	
In proximity to primary care				
Not in proximity to primary care	38.5 (408/1060)	5.3 (50/936)	1.00	<0.001
In proximity to primary care	61.5 (652/1060)	94.7 (886/936)	11.01 (8.13–15.13)	
Regular primary care				
Not on regular primary care followup	18.1 (192/1060)	47.8 (447/936)	1.00	<0.001
On regular primary care followup	81.9 (868/1060)	52.2 (489/936)	0.24 (0.20–0.30)	
Demographic characteristics				
Age				
< 60 years	47.3 (501/1060)	49.5 (463/936)	1.00	0.346
≥ 60 years	52.7 (559/1060)	50.5 (473/936)	0.92 (0.77–1.09)	
Ethnicity				
Non-Chinese	23.8 (252/1060)	47.0 (440/936)	1.00	<0.001
Chinese	76.2 (808/1060)	53.0 (496/936)	0.35 (0.29–0.43)	
Marital status				
Not married	29.8 (316/1060)	53.0 (496/936)	1.00	<0.001
Married	70.2 (744/1060)	47.0 (440/936)	0.38 (0.31–0.45)	
Gender				
Female	59.5 (631/1060)	55.9 (523/936)	1.00	0.102
Male	40.5 (429/1060)	44.1 (413/936)	1.16 (0.97–1.39)	
Socioeconomic characteristics				
Occupation				
Unemployed	58.7 (622/1060)	62.4 (584/936)	1.00	0.099
Employed	41.3 (438/1060)	37.6 (352/936)	0.86 (0.72–1.03)	
Financial aid				
Not on financial aid	93.0 (986/1060)	81.6 (764/936)	1.00	<0.001
On financial aid	7.0 (74/1060)	18.4 (172/936)	3.00 (2.25–4.00)	
Monthly household income				
≤ $500	13.2 (140/1060)	31.9 (299/936)	1.00	
≥ $500, <$1000	11.5 (122/1060)	63.7 (596/936)	2.29 (1.73–3.03)	<0.001
≥ $1000	75.3 (798/1060)	4.4 (41/936)	0.02 (0..02–0.04)	
Education				
Primary and below	37.7 (400/1060)	74.8 (700/936)	1.00	<0.001
Secondary	34.6 (367/1060)	22.9 (214/936)	0.33 (0.27–0.41)	
Tertiary	27.6 (293/1060)	2.4 (22/936)	0.04 (0.03–0.07)	
Medical characteristics				
Charlson Comorbidity Index (CCMI)				
CCMI = 0	80.3 (851/1060)	68.1 (637/936)	1.00	<0.001
CCMI > 0	19.7 (209/1060)	31.9 (299/936)	1.91 (1.56–2.35)	
Chronic pain (pain ≥ 3 months)				
No chronic pain	85.7 (908/1060)	85.8 (803/936)	1.00	0.949
Chronic pain	14.3 (152/1060)	14.2 (133/936)	0.99 (0.77–1.27)	
Hypertension				
No hypertension	60.0 (636/1060)	61.4 (575/936)	1.00	0.521
Has hypertension	40.0 (424/1060)	38.6 (361/936)	0.94 (0.79–1.13)	
Diabetes				
No diabetes	85.8 (909/1060)	79.5 (744/936)	1.00	<0.001
Has diabetes	14.2 (151/1060)	20.5 (192/936)	1.55 (1.23–1.96)	
Hyperlipidemia				
No hyperlipidemia	62.8 (666/1060)	68.5 (641/936)	1.00	0.008
Has hyperlipidemia	37.2 (394/1060)	31.5 (295/936)	0.78 (0.65–0.94)	
Overweight				
Not overweight	56.4 (594/1054)	54.7 (509/930)	1.00	0.469
Overweight	43.6 (460/1054)	45.3 (421/930)	1.07 (0.89–1.28)	

### Association between usage of subsidized primary care, proximity to primary care, and regular primary care with regular participation in cardiovascular and cancer screening

In the rental flat population, for cardiovascular screening, regular primary care was independently associated with regular diabetes screening (adjusted odds ratio, aOR = 1.59, CI = 1.12–2.26, *p* = 0.009) and hyperlipidemia screening (aOR = 1.82, CI = 1.10–3.04, *p* = 0.023). In the owner-occupied flats, regular primary care was independently associated with regular hypertension screening (aOR = 9.34 (1.82–47.85, *p* = 0.007), while usage of subsidized primary care was associated with regular diabetes screening (aOR = 2.94, CI = 1.04–8.31, *p* = 0.042) (Table 3).

**Table 3:** Association between primary care characteristics and health screening participation in low socioeconomic status and higher socioeconomic status neighborhoods

Rental flat (low- SES) population, hypertension screening (*N* = 575)						Non-rental flat (higher-SES) population, hypertension screening (*N* = 614)					
Hypertension screening	Going for regular screening, *N* (%)	OR (95 % CI)	*p*-value	aOR^a^ (95 % CI)	*p*-value	Hypertension screening	Going for regular screening, *N* (%)	OR (95 % CI)	*p*-value	aOR^a^ (95 % CI)	*p*-value
Not on subsidized primary care (CHAS scheme)	45.1 (125/277)	1.00	0.737	1.00	0.949	Not on subsidized primary care (CHAS scheme)	57.9 (310/535)	1.00	0.181	1.00	0.503
On subsidized primary care (CHAS scheme)	43.6 (130/298)	0.94 (0.68–1.31)		0.99 (0.66–1.47)		On subsidized primary care (CHAS scheme)	49.4 (39/79)	0.71 (0.44–1.14)		0.76 (0.33–1.71)	
Not in proximity to primary care	53.3 (16/30)	1.00	0.348	1.00	0.465	Not in proximity to primary care	60.3 (167/277)	1.00	0.121	1.00	0.258
In proximity to primary care	43.9 (239/545)	0.68 (0.33–1.43)		0.73 (0.32–1.70)		In proximity to primary care	54.0 (182/337)	0.77 (0.56–1.07)		0.80 (0.55–1.18)	
Not on regular primary care followup	39.5 (107/271)	1.00	0.029	1.00	0.172	Not on regular primary care followup	40.6 (26/64)	1.00	0.007	1.00	0.007
On regular primary care followup	48.7 (148/304)	1.45 (1.04–2.03)		1.29 (0.90–1.85)		On regular primary care followup	58.7 (323/550)	2.08 (1.23–3.52)		9.34 (1.82–47.85)	
Rental flat (low- SES) population, diabetes screening (*N* = 744)						Non-rental flat (higher-SES) population, diabetes screening (*N* = 887)					
Diabetes screening	Going for regular screening, *N* (%)	OR (95 % CI)	*p*-value	aOR^b^ (95 % CI)	*p*-value	Diabetes screening	Going for regular screening, *N* (%)	OR (95 % CI)	*p*-value	aOR^b^ (95 % CI)	*p*-value
Not on subsidized primary care (CHAS scheme)	49.9 (183/367)	1.00	0.005	1.00	0.080	Not on subsidized primary care (CHAS scheme)	57.4 (413/720)	1.00	0.015	1.00	0.042
On subsidized primary care (CHAS scheme)	39.5 (149/377)	0.66 (0.49–0.88)		0.74 (0.53–1.04)		On subsidized primary care (CHAS scheme)	67.7 (113/167)	1.56 (1.09–2.22)		2.94 (1.04–8.31)	
Not in proximity to primary care	41.5 (17/41)	1.00	0.748	1.00	0.083	Not in proximity to primary care	54.4 (193/355)	1.00	0.015	1.00	0.558
In proximity to primary care	44.8 (315/703)	1.15 (0.61–2.17)		1.87 (092–3.78)		In proximity to primary care	62.6 (333/532)	1.41 (1.07–1.85)		1.10 (0.79–1.54)	
Not on regular primary care followup	37.6 (130/346)	1.00	<0.001	1.00	0.009	Not on regular primary care followup	66.0 (95/144)	1.00	0.079	1.00	0.079
On regular primary care followup	50.8 (202/398)	1.71 (1.28–2.30)		1.59 (1.12–2.26)		On regular primary care followup	58.0 (431/743)	0.71 (0.49–1.04)		0.37 (0.12–1.12)	
Rental flat (low- SES) population, hyperlipidemia screening (*N* = 640)						Non-rental flat (higher-SES) population, hyperlipidemia screening (*N* = 643)					
Hyperlipidemia screening	Going for regular screening, *N* (%)	OR (95 % CI)	*p*-value	aOR (95 % CI)^c^	*p*-value	Hyperlipidemia screening	Going for regular screening, *N* (%)	OR (95 % CI)	*p*-value	aOR^c^ (95 % CI)	*p*-value
Not on subsidized primary care (CHAS scheme)	37.6 (115/306)	1.00	0.213	1.00	0.931	Not on subsidized primary care (CHAS scheme)	46.9 (238/507)	1.00	0.053	1.00	0.691
On subsidized primary care (CHAS scheme)	32.6 (109/334)	0.81 (0.59–1.11)		0.98 (0.67–1.45)		On subsidized primary care (CHAS scheme)	56.6 (77/136)	1.48 (1.01–2.16)		1.22 (0.45–3.30)	
Not in proximity to primary care	41.4 (12/29)	1.00	0.550	1.00	0.770	Not in proximity to primary care	44.6 (108/242)	1.00	0.088	1.00	0.825
In proximity to primary care	34.7 (212/611)	0.75 (0.35–1.61)		0.88 (0.39–2.01)		In proximity to primary care	51.6 (207/401)	1.32 (0.96–1.82)		1.04 (0.71–1.52)	
Not on regular primary care followup	28.2 (91/323)	1.00	<0.001	1.00	0.023	Not on regular primary care followup	56.8 (67/118)	1.00	0.067	1.00	0.569
On regular primary care followup	42.0 (133/317)	1.84 (1.33–2.56)		1.82 (1.10–3.04)		On regular primary care followup	47.2 (248/525)	0.68 (0.46–1.02)		0.68 (0.19–2.53)	
Rental population (low- SES), colorectal cancer screening (*N* = 722)						Non-rental flat (higher-SES) population, colorectal cancer screening (*N* = 866)					
FOBT screening	Going for regular screening, *N* (%)	OR (95 % CI)	*p*-value	aOR (95 % CI)^d^	*p*-value	FOBT screening	Going for regular screening, *N* (%)	OR (95 % CI)	*p*-value	aOR (95 % CI)^d^	*p*-value
Not on subsidized primary care (CHAS scheme)	8.7 (30/346)	1.00	0.788	1.00	0.810	Not on subsidized primary care (CHAS scheme)	18.9 (129/682)	1.00	0.016	1.00	0.348
On subsidized primary care (CHAS scheme)	8.0 (30/376)	0.91 (0.54–1.55)		0.93 (0.49–1.74)		On subsidized primary care (CHAS scheme)	11.4 (21/184)	0.55 (0.34–0.90)		0.55 (0.15–1.94)	
Not in proximity to primary care	18.2 (8/44)	1.00	0.023	1.00	0.049	Not in proximity to primary care	15.9 (56/352)	1.00	0.411	1.00	0.049
In proximity to primary care	7.7 (52/678)	0.37 (0.17–0.85)		0.42 (0.17–0.99)		In proximity to primary care	18.3 (94/514)	1.18 (0.82–1.70)		1.48 (1.01–2.21)	
Not on regular primary care followup	7.1 (24/337)	1.00	0.344	1.00	0.450	Not on regular primary care followup	11.1 (18/162)	1.00	0.021	1.00	0.847
On regular primary care followup	9.4 (36/385)	1.35 (0.79–2.31)		1.28 (0.67–2.45)		On regular primary care followup	18.8 (132/704)	1.85 (1.09–3.12)		1.14 (0.29–4.54)	
Rental population (low- SES), cervical cancer screening (*N* = 334)						Non-rental flat (higher-SES) population, cervical cancer screening (*N* = 421)					
Pap smear screening	Going for regular screening (*N* %)	OR (95 % CI)	*p*-value	aOR^e^ (95 % CI)	*p*-value	Pap smear screening	Going for regular screening (*N* %)	OR (95 % CI)	*p*-value	aOR^e^ (95 % CI)	*p*-value
Not on subsidized primary care (CHAS scheme)	16.3 (24/147)	1.00	0.566	1.00	0.372	Not on subsidized primary care (CHAS scheme)	26.0 (81/312)	1.00	0.001	1.00	0.047
On subsidized primary care (CHAS scheme)	19.3 (36/187)	1.22 (0.69–2.16)		2.69 (0.68–2.78)		On subsidized primary care (CHAS scheme)	43.1 (47/109)	2.16 (1.37–3.41)		7.93 (1.03–62.51)	
Not in proximity to primary care	14.3 (2/14)	1.00	1.00	1.00	0.795	Not in proximity to primary care	14.7 (21/143)	1.00	<0.001	1.00	<0.001
In proximity to primary care	18.1 (58/320)	1.33 (0.29–6.10)		1.24 (0.25–6.29)		In proximity to primary care	38.5 (107/278)	3.64 (2.16–6.13)		3.22 (1.72–5.84)	
Not on regular primary care followup	14.6 (23/157)	1.00	0.154	1.00	0.394	Not on regular primary care followup	42.3 (44/84)	1.00	0.003	1.00	0.750
On regular primary care followup	20.9 (37/177)	1.54 (0.87–2.73)		1.49 (0.86–3.77)		On regular primary care followup	26.5 (84/317)	0.49 (0.31–0.78)		0.65 (0.04–9.52)	
Rental population (low- SES), breast cancer screening (*N* = 517)						Non-rental flat (higher-SES) population, breast cancer screening (*N* = 609)					
Mammogram screening	Going for regular screening (*N* %)	OR (95 % CI)	*p*-value	aOR^e^ (95 % CI)	*p*-value	Mammogram screening	Going for regular screening (*N* %)	OR (95 % CI)	*p*-value	aOR^e^ (95 % CI)	*p*-value
Not on subsidized primary care (CHAS scheme)	10.2 (24/236)	1.00	0.053	1.00	0.009	Not on subsidized primary care (CHAS scheme)	9.8 (46/469)	1.00	0.001	1.00	0.006
On subsidized primary care (CHAS scheme)	16.0 (45/281)	1.68 (0.99–2.86)		2.33 (1.23–4.41)		On subsidized primary care (CHAS scheme)	21.4 (30/140)	2.51 (1.51–4.16)		6.02 (1.69–21.28)	
Not in proximity to primary care	25.0 (6/24)	1.00	0.115	1.00	0.023	Not in proximity to primary care	6.6 (16/244)	1.00	<0.001	1.00	0.032
In proximity to primary care	12.8 (63/493)	0.44 (0.17–1.15)		0.29 (0.10–0.84)		In proximity to primary care	16.4 (60/365)	2.80 (1.57–4.99)		2.22 (1.08–4.54)	
Not on regular primary care followup	12.1 (31/257)	1.00	0.439	1.00	0.855	Not on regular primary care followup	19.7 (25/127)	1.00	0.010	1.00	0.025
On regular primary care followup	14.6 (38/260)	1.25 (0.75–2.08)		1.08 (0.48–2.42)		On regular primary care followup	10.6 (51/482)	0.48 (0.29–0.82)		0.10 (0.01–0.75)	

^a^Controlling for maturity of housing estate, ethnicity, marital status, gender, financial aid, education level, and comorbidities, diabetes and hyperlipidemia in multivariate clustered logistic regression model

^b^Controlling for maturity of housing estate, age, gender, education level, hypertension and hyperlipidemia in multivariate clustered logistic regression model

^c^Controlling for age, marital status, employment, household income, financial aid, comorbidities, and hypertension in multivariate clustered logistic regression model

^d^Controlling for ethnicity, marital status, employment, education level, and comorbidities in multivariate clustered logistic regression model

^e^Controlling for maturity of housing estate, age, ethnicity, marital status, employment, household income, financial aid, education level, and comorbidities in multivariate clustered logistic regression model

For cancer screening, in the rental flat population, proximity to primary care was associated with less participation in regular colorectal cancer screening through fecal occult blood testing (aOR = 0.42, CI = 0.17–0.99, *p* = 0.049); and less participation in regular breast cancer screening through mammography (aOR = 0.29, CI = 0.10–0.84, *p* = 0.023). Usage of subsidized primary care (being on the CHAS scheme) was only associated with increased participation in regular mammography (aOR = 2.33, CI = 1.23–4.41, *p* = 0.009). In the owner-occupied flat population, proximity to primary care was associated with higher participation in colorectal cancer screening (aOR = 1.48, CI = 1.01 = 2.21, *p* = 0.049). For gynecological cancer screening in the owner-occupied flat communities, a consistent pattern emerged. Usage of subsidized primary care and proximity to primary care was associated with higher rates of breast cancer and cervical cancer screening; however, being on regular primary care followup was associated with lower rates of mammography (aOR = 0.10, CI = 0.01–0.75, *p* = 0.025).

### Barriers to cancer and cardiovascular screening on qualitative analysis

There were a total of 20 patient participants. All came from the low-SES public rental flat neighborhoods. The majority were Chinese (85 %). These patients were of lower-SES: two-thirds were unemployed, and all had a household income of ≤ $1500/month (compared with the average household income of S$2380) [23].

### Major content areas

For the screening modalities (hypertension, diabetes, and dyslipidemia), patient comments fell into several content areas: primary care characteristics, knowledge, priorities and attitudes. Representative quotations of the various content areas are presented in Additional file 2: Table S2 (cancer screening) and Additional file 3: Table S3 (cardiovascular screening).

### Barriers to screening in primary care

Certain characteristics of primary care emerged consistently as barriers to both cancer and cardiovascular screening. In particular, long waiting times at the public polyclinic, distance to the polyclinic and inconvenient opening hours were commonly cited barriers:

Yes, it is very inconvenient (to go for screening) because I go to the polyclinic which is quite a distance away. And the polyclinic isn’t open on weekends and only open for half the day on Friday. So it’s difficult for me to take leave from work to go. (Hyperlipidemia screening)

In addition, the doctor-patient relationship and patient interaction was also important in encouraging screening. For both cancer and cardiovascular screening, patients were put off by hurried consultations, or discouraged by a lack of trust between them and the doctor.

Sometimes the doctor talks about high blood pressure and diabetes, but I’m not too sure what he’s talking about. Don’t know, doctor never explain. He’s in a rush, just listen to my heart, says everything is ok. Everything is ok, then I don’t want to ask so much also. Don’t want to waste the doctor’s time. (Hypertension screening)

For cancer screening, embarrassment and discomfort with the screening procedure surfaced as reasons why patients might avoid screening at primary care:

They will stick up a stick into the vagina and scrape- so embarrassing! And awkward. I don’t know how to ask for it. It is difficult. Especially with a male doctor, even more difficult. (Pap smear)

### Patient knowledge, priorities and attitudes as barriers to screening

Knowledge, priorities and attitudes also featured as important barriers to cancer and cardiovascular screening. In particular, fear of diagnosis and a fatalistic attitude that nothing could be done even if disease was detected was a key reason why screening was put off or delayed until the last possible moment.

I rarely go to the doctor and I’m very scared too. I haven’t seen the doctor since I was young. Once we check and find out we have illnesses, we would worry a lot. Not knowing is better. (Colorectal cancer screening)

In addition, in this low-SES population, pressures of work and other priorities was an additional reason why screening was delayed.

I have a cousin who died of cervical cancer who didn’t go for treatment because she was busy working. It’s sad but same here, I’ve to keep my job. I can’t afford to take time off for screening. (Cervical cancer screening)

Finally, patients did not buy into the concept of early detection and prevention; they believed that disease only begins with the onset of symptoms. In these cases, while they were definitely keen to see a doctor in the event that they did not feel well or had physical symptoms, they were not keen to consult a doctor when they had no physical symptoms.

If it (blood sugar) was slightly high, I don’t really see a need to go see a doctor. Unless there is clear evidence that it is very high and requires medications then I would go. If the illness hasn’t appeared I don’t have the urge to see the doctor. I know it’s good to discover it early but that’s not enough to urge me to go. (Diabetes screening)

One will know if one is healthy or unhealthy. If you ache all over, you definitely have to go see the doctor! If not, why need! When I don’t feel well, then I will go. (Hyperlipidemia screening)

## Discussion

Having regular primary care was independently associated with regular participation in cardiovascular screening for hypertension, diabetes and hyperlipidemia, in both rental flat (low-SES) populations as well as owner-occupied (higher-SES) flat communities. Surprisingly, usage of subsidized primary care was independently associated with regular diabetes screening in the owner-occupied flat population, but not in the rental flat population, after controlling for individual-SES and other sociodemographic factors. This suggests the importance of the doctor-patient relationship in encouraging regular cardiovascular screening, even amongst low-SES populations. Our previous studies showed that only a small minority (~10 %) of residents in public rental flat neighborhoods in Singapore preferred to approach Western-trained physicians for primary care. In particular, social distance between the medical practitioner and the patient, as well as a marked preference for self-reliance for “minor ailments”, only consulting in the presence of symptoms or medical emergencies, were highlighted as barriers to consulting Western-trained primary physicians [16]. In terms of social distance, studies have shown that socioeconomic status can influence doctor-patient communication [26]. Patients from lower social classes receive less positive socio-emotional utterances and a more directive and less participatory consulting style, characterized by less information giving, less directions and less socio-emotional and partnership building utterances from their doctor. Encouraging greater continuity of care by allowing lower-income Singaporeans to receive subsidized primary care via the CHAS program from private GPs (as opposed to public polyclinics, with lesser physician continuity as the doctor rotates) [27] may thus help to improve continuity of care. This is particularly relevant for cardiovascular screening. Our previous research suggests that providing free screening interventions in low-income communities is insufficient, by itself, to improve screening rates amongst those most in need of intervention [18]- this is because patients take into account not just the cost of screening, but also the cost of treatment (if they were to have a positive diagnosis). A better doctor-patient relationship can potentially provide the additional “nudge” to go for screening; these hypotheses are supported by the results of our qualitative study, which demonstrated that patients were discouraged from participating in screening by rushed consultations, or by a lack of trust in the doctor-patient relationship. Healthy patient-doctor relationships were also cited as important factors in other urbanized Asian societies [28].

Interestingly, a different picture emerged for cancer screening. For the low-income community, proximity to primary care was associated with less participation in regular colorectal cancer screening and breast cancer screening; compared with the owner-occupied community, in which greater proximity was associated with regular mammography. For the owner-occupied population, regular primary care was associated with lower participation in mammography screening. We offer two possible explanations for these findings. Residents of urbanized low-SES areas have a higher tendency for out-of-hours and unscheduled use of primary care [29], compared to their counterparts living in more affluent neighborhoods [30]. Perhaps for those staying in close proximity to polyclinic, their resistance to seeking medical consultation is higher because staying adjacent to the polyclinic reassures them that they can seek medical consult promptly should symptoms manifest. This in turn translates into lower participation in screening with greater proximity to the public polyclinic. This was supported by the findings of our qualitative study, in which residents in the public rental neighbourhood acknowledged that they would seek medical consult in the event of symptoms or emergencies, but otherwise were keen to minimize their contact with primary care. Studies with other disadvantaged populations also identified postponing of consultation as a coping mechanism [31]. Alternatively, in our Asian society, embarrassment regarding cancer screening (privacy concerns for gynaecological cancer screening, and revulsion regarding handling of fecal material) could discourage patients from seeking screening because of concerns over “losing face”. Thus, in the low-SES area, where patients were less mobile, greater proximity to primary care was associated with less regular cancer screening because they were afraid of “losing face” in the neighbourhood. On the other hand, in the higher-SES area, as members of the higher-SES population already have access to additional resources (e.g. private non-subsidised GPS, company doctors, etc) outside the neighbourhood, their main concern was not so much fear of embarrassment within the neighbourhood, but fear of embarrassment at the doctor’s office- they found it difficult and awkward to bring up the conversation about screening, especially with doctors that were seeing them on a regular basis.

The limitations of our study are as follows. Our study was a cross-sectional one; thus we can only conclude correlation, not causation, between primary care characteristics and health screening. In addition, we only covered five public housing estates in Singapore; we were unable to do a nation-wide survey of the public rental flat population due to logistical difficulties, as public rental flat enclaves are scattered across the entire island. However, the sociodemographic charcteristics of our population were broadly similar to national data on the public rental flat population. In our measures of proximity, we only investigated physical distance - we did not account for other factors like journey times. In densely populated urban Singapore, with generally short point-to-point distances, journey time and distances are unlikely to vary by much. Finally, we did not investigate other characteristics of primary care, such as practice ownership in our study.

## Conclusion

Having regular primary care was independently associated with regular participation in cardiovascular screening for hypertension, diabetes and hyperlipidemia, in both rental flat (low-SES) populations as well as owner-occupied (higher-SES) flat communities. This suggests that the doctor-patient relationship is important for encouraging regular cardiovascular screening; in addition, it may indicate that for cardiovascular disease screening does not stand in isolation- the screening conversation needs to bear in mind implications of diagnosis and treatment. Conversely for cancer screening, in the low-SES community, proximity to primary care was associated with less participation in regular colorectal cancer screening and breast cancer screening, while in the higher-SES population, regular primary care was associated with lower participation in mammography screening. In the Asian context, this may be due to embarrassment and awkwardness about cancer screening, with fear of “losing face” before neighbors, relatives and friends predominant in the low SES community, and fear of embarrassment before their regular doctor predominant in the higher-SES community. These factors should be taken into account when attempting to intervene in disadvantaged populations to address disparities in access to primary care, particularly in rapidly urbanising Asian societies.

## Additional files

Additional file 1: Table S1. Qualitative interview guide for residents. (DOCX 14 kb) Additional file 2: Table S2. Representative quotes from patients staying in a public rental flat neighborhood on barriers to cancer screening, organized by frequently mentioned content areas and themes. (DOCX 18 kb) Additional file 3: Table S3. Representative quotes from patients staying in a public rental flat neighborhood on barriers to cardiovascular screening, organized by frequently mentioned content areas and themes. (DOCX 18 kb)
