# Supplementary material for: Primary care characteristics and their association with health screening in a low-socioeconomic status public rental-flat population in Singapore- a mixed methods study
Source: BMC Fam Pract. 2016 Feb 6;17:16. doi: 10.1186/s12875-016-0411-5 (PMC4744417; doi:10.1186/s12875-016-0411-5)
Supplement: Additional file 1: Table S1. — Qualitative interview guide for residents. (DOCX 14 kb) [file 12875_2016_411_MOESM1_ESM.docx]

**Supplementary Table 1.** Qualitative interview guide for residents

| ***Section A: Specific screening modality questions for residents*** | |
| --- | --- |
| **Hypertension** | **Colorectal cancer:** |
| 1. Have you ever heard of a test for hypertension (blood pressure measurement using sphygmomanometer)?  2. What do you feel/think about this test?  3. What might keep you from doing this test? What makes it difficult for you to do this test?  4. What kinds of things would work to get people, like yourself, to want to do this test? What makes it easy for you to do this test? | 1. Have you ever heard of a test for colorectal cancer to look for blood in the stool?  2. What do you feel/think about this test?  3. What might keep you from doing this ‘‘blood stool test’? What makes it difficult for you to do this test?  4. What kinds of things would work to get people, like yourselves, to want to do the blood stool test? What makes it easy for you to do this test? |
| **Diabetes** | **Cervical cancer:** |
| 1. Have you ever heard of a test for diabetes (fasting blood glucose test)?  2. What do you feel/think about this test?  3. What might keep you from doing this test? What makes it difficult for you to do this test?  4. What kinds of things would work to get people, like yourself, to want to do this test? What makes it easy for you to do this test? | 1. Have you ever heard of a test for cervical cancer?  2. What do you feel/think about this test?  3. What might keep you from doing the pap smear? What makes it difficult for you to do this test?  4. What kinds of things would work to get people, like yourselves, to want to do pap smears? What makes it easy for you to do this test? |
| **Dyslipidemia** | **Breast cancer:** |
| 1. Have you ever heard of a test for high cholesterol (fasting blood lipid test)?  2. What do you feel/think about this test?  3. What might keep you from doing this test? What makes it difficult for you to do this test?  4. What kinds of things would work to get people, like yourself, to want to do this test? What makes it easy for you to do this test? | 1.Have you ever heard of a test for breast cancer?  2. What do you feel/think about this test?  3. What might keep you from doing mammograms? What makes it difficult for you to do this test?  4. What kinds of things would work to get people, like yourselves, to want to do mammograms? What makes it easy for you to do this test? |
