# Supplementary material for: Primary care characteristics and their association with health screening in a low-socioeconomic status public rental-flat population in Singapore- a mixed methods study
Source: BMC Fam Pract. 2016 Feb 6;17:16. doi: 10.1186/s12875-016-0411-5 (PMC4744417; doi:10.1186/s12875-016-0411-5)
Supplement: Additional file 2: Table S2. — Representative quotes from patients staying in a public rental flat neighborhood on barriers to cancer screening, organized by frequently mentioned content areas and themes. (DOCX 18 kb) [file 12875_2016_411_MOESM2_ESM.docx]

**Supplementary Table 2.** Representative quotes from patients staying in a public rental flat neighborhood on barriers to cancer screening, organized by frequently mentioned content areas and themes

| **Primary care characteristics** | |
| --- | --- |
| *Barriers* | |
| **Lack of trust in healthcare system/healthcare professionals** | “The doctor didn’t really explain clearly to me why I needed the test. And he said that I might need to do a scope after that- so I asked him whether this test was good enough, if it’s normal, does that mean that I won’t have any problems for at least a year? He didn’t really give me a straightforward answer. I wonder whether he really knows what he’s doing or whether he just wants me to come back more often so he can charge more.” (CRC) |
| **Healthcare professional does not often discuss screening – no time** | “The last time I went to the clinic I wanted to ask about the mammogram but the doctor was in a rush and just kept hurrying me so I didn’t ask in the end.” (BC) |
| **Embarrassment about screening modality** | “They will stick up a stick into the vagina and scrape- so embarrassing! And awkward. I don’t know how to ask for it. It is difficult. Especially with a male doctor, even more difficult.” (CC)  “It (FOBT screening) is so dirty! So *malu^a^*, how to do?” (CRC) |
| **Characteristics of clinic (manpower, location, hours open)** | “But I think it is quite troublesome. I heard that you have to book an appointment. And I heard it’s quite hard to schedule appointment, it is by first come first serve. And the queue is always long.” (CC)  “It’s quite far and there’s, no one to take me. My daughter stays very far away. I don’t want to trouble her too often. The other children are working, and on Sunday the polyclinic’s closed. It’s also closed in the evenings so they can’t bring me.” (BC) |
| **Knowledge** | |
| *Barriers* | |
| **Not aware of screening** | “No one told me about it. People every time say ‘cancer, cancer’, but I don’t know what kind of illness it is. I don't know about screening. Never heard of it. If you didn’t tell me about this test, I wouldn’t have known also.” (CRC) |
| **No need screening as healthy /not at risk** | “There is nothing wrong with me. I don’t have any illnesses. I am healthy. I would not go. There is nothing wrong with me so I don’t need to do the test. I am okay.” (CRC)  “I’m not sick, how am I to go? Must be sick first. If I am sick, then I’ll go. If I’m not sick, then not sick what. If I am sick, I want to check. But the thing is that I am not sick, I already said. How do you expect me to go and see a doctor?” (CC)  “So if I discover a lump, I might then go to see the doctor. I won't go for screenings or see the doctor unless it's a major illness with symptoms. Since I’ve no symptoms, I don't want to go.” (BC) |
| **Not aware of where to go for screening** | “Where is Singapore Cancer Society? Where can I get the kit? From the polyclinic? I don’t know where to get the kit so I’ve never used it.” (CRC |
| **Screening may not be accurate/ alternative screening methods are better** | “I do regular self-examinations and check if there are lumps. No need for mammogram. Cheaper, more accurate too, I can feel lumps the size of green beans.” (BC) |
| **Last test normal, so no need to go again** | “But now my feces are normal, I know it. The last time I tested, no blood. So I never went again because everything is ok.” (CRC) |
| **Confusion that mammogram causes cancer** | “I think when you take mammogram, the pressure on my breasts, there can be inflammation inside which can lead to cancer. Also when I was screening I saw the radiographer taking cover behind a shield, they cannot be exposed to too much radiation. I became worried about radiation after that.” (BC) |
| **Priorities** | |
| *Barriers* | |
| **No time to go, too busy** | “That day I went to see the doctor, he asked me to do, but I said I’m working, I can't. I've been with this company less than a month, if suddenly there something, my employer will be scared.” (CRC)  “I have a cousin who died of cervical cancer who didn't go for treatment because she was busy working. It’s sad but same here, I’ve to keep my job. I can’t afford to take time off for screening.” (CC)  “No, because of time and we housewives are very busy with housework and the kids. How to go for screening?” (BC) |
| **Can spend money on other things** | “If it was free of course I would be even more willing. I can save $50. I can buy a lot of things with $50. So many other things to buy, like groceries, school books, how to spend on the test?” (CC) |
| **Attitudes** | |
| *Barriers* | |
| **Fatalism** | “It can’t be helped. Even if you don’t smoke, also can get cancer. I’m not afraid! Never mind, if I have to die I have to die, nothing tough about it.” (CRC) |
| **Fear of diagnosis and/or treatment** | “I rarely go to the doctor and I'm very scared too. I haven’t seen the doctor since I was young. Once we check and find out we have illnesses, we would worry a lot. Not knowing is better.” (CRC)  “Yes, that is one of my reasons. So that's why I'm scared of going to the hospital. With all their tests, their reports might show that I have some illness. Yes, but if you don't go you wouldn't hear such bad news. Let's say they tell you (that) you only have a few days left, I still have my two young kids so I worry about this. I'm most afraid of being diagnosed with an illness.” (CC)  “And if I go for screening and am diagnosed, I will still need long term treatment. If there is fast remedy maybe I don’t mind. Because my uncle had cancer, went to treatment and in the end, there was no use at all. I heard chemo and radiotherapy is very painful and your breasts will turn black.” (BC) |
| **Too old to go for screening** | “I don’t think I need this test because I am old, it doesn’t matter anymore.” (CRC) |
| **Traditional medicine is better** | “I heard from my friends that *sinseh*^b^ have some method of sucking the cancer out and afterwards the patient can recover. So if the *sinseh*^b^ is better then I should go to the *sinseh*^b^ to checkup, why should I go to the western doctor?” (BC) |
| **Disease not important** | “Other people did mention about colorectal cancer but I didn’t really care about it. I think it’s not important to me.” (CRC) |

CRC: Quotations from patients in reference to colorectal cancer screening using fecal occult blood testing

CC: Quotations from patients in reference to cervical cancer screening using pap smears

BC: Quotations from patients in reference to breast cancer screening using mammograms

^a^ *Malu*: Malay for embarrassed

^b^ *Sinseh*: traditional Chinese medicine practitioner
