# Supplementary material for: Primary care characteristics and their association with health screening in a low-socioeconomic status public rental-flat population in Singapore- a mixed methods study
Source: BMC Fam Pract. 2016 Feb 6;17:16. doi: 10.1186/s12875-016-0411-5 (PMC4744417; doi:10.1186/s12875-016-0411-5)
Supplement: Additional file 3: Table S3. — Representative quotes from patients staying in a public rental flat neighborhood on barriers to cardiovascular screening, organized by frequently mentioned content areas and themes. (DOCX 18 kb) [file 12875_2016_411_MOESM3_ESM.docx]

**Supplementary Table 3.** Representative quotes from patients staying in a public rental flat neighborhood on barriers to cardiovascular screening, organized by frequently mentioned content areas and themes

| **Primary care characteristics** | |
| --- | --- |
| *Barriers* | |
| **Lack of trust in healthcare system/healthcare professionals** | “I rarely see doctors. Don’t want. I never liked to see doctors all along. Since young I never liked to see doctors. I don’t trust them. Every time I go polyclinic, I see a different one. How does he know what’s going on? And the nurses too. The other day, I went for some blood test. There was a foreign nurse. Taking blood is not meant to be painful, but she punctured all the way to my bone or something. I told her to take the needle out. I’m not going to go for a blood test again.”(DM) |
| **Healthcare professional does not often discuss screening – no time** | “Sometimes the doctor talks about high blood pressure and diabetes, but I’m not too sure what he’s talking about. Don’t know, doctor never explain. He’s in a rush, just listen to my heart, say everything is ok. Everything is ok, then I don’t want to ask so much also. Don’t want to waste the doctor’s time. If everything is ok, then ok.” (HTN) |
| **Characteristics of clinic (manpower, location, hours open)** | “No. I don’t want to go. Because the waiting times at the polyclinics are very long. At least, if you include collecting medication, the fastest is about 1 hour. It’s far and I have to take a bus all the way to the polyclinic to see a doctor, not very easy to go.” (HTN)  “The polyclinic doesn’t open when I’m free on Sundays. I’m just very scared of queuing and waiting. It’s very frustrating. Have to wait really long, I’m very scared.” (DM)  “Yes, it is very inconvenient because I go to the polyclinic which is quite a distance away. And the polyclinic isn't open on weekends and only open for half the day on Friday. So it’s difficult for me to take leave from work to go.” (HChol) |
| **Knowledge** | |
| *Barriers* | |
| **Not aware of screening** | “I am not sure. Because I roughly know about high blood pressure, what it is and the complications like heart problems and stroke if it’s too high. But cholesterol I don't know. I don’t know how to test for it also.” (HChol)  “Yes I have heard of diabetes, but I don’t know how to test for it. I am not knowledgeable about all these diseases.” (DM) |
| **No need screening as healthy** | “I am not sick, there is no reason why I should check. If I start feeling very tired, then go check. I think I’m ok, steady, heart’s ok. Don’t care about high blood and cholesterol, all small things. I’m healthy.” (HTN)  “If it (blood sugar) was slightly high, I don't really see a need to go see a doctor. Unless there is clear evidence that it is very high and requires medications then I would go. Because if the illness hasn't appeared I don't have the urge to see the doctor. I know it's good to discover it early but that's not enough to urge me to go.” (DM)  “I guess we are healthy if we are not sick. Actually, one will know if one is healthy or unhealthy. If you ache all over, you definitely have to go see the doctor! If not, why need! When I don't feel well, then I will go.” (HChol) |
| **No need screening as not at risk** | “If a person has diabetes you can see it. Look at the person’s face, you can see if the person has a problem. If the person’s appearance doesn’t look very well then you know that the person has an illness. I have never had that problem, I look well. I won’t get diabetes, my father and mother never had diabetes, none of my siblings have, I eat healthily, so I’ll be ok.” (DM)  “I know I don’t have high cholesterol. Because I hardly ever eat fried stuff. And also because I’m so skinny. For those things that I can’t possibly have, I won't be scared of them and I won't go and test for them.” (HChol) |
| **Not aware of where to go for screening** | “I know it’s an important thing, but I don’t know where to get the test. Do you need to make an appointment?” (DM) |
| **Last test normal, so no need to go again** | “The last time I tested was 5 years ago, at that time doctor said everything was fine, no problem. That’s why I never went again, because I know these things take some time to develop, so I think I can afford to wait, see how.” ((HChol) |
| **Screening may not be accurate/ alternative screening methods are better** | “Actually diabetes is very easy to test! No need blood test, you can just test at home. My friends tell me, if's there's glucose in the urine, there will be ants and you will know. Diabetes- you have to test urine right? They didn’t test my urine. So I didn’t go.” (DM) |
| **Priorities** | |
| *Barriers* | |
| **No time to go, too busy** | “I have to work. Even on Saturday I still have to work half-day. So I can’t take leave to go. Even if I have something, there’s no time to see the doctor anyway.” (HTN)  “No I didn’t go for screening. I didn’t care. Because I work, I’m busy thinking of work. Don’t want to waste time. If you have time it’s okay, but when you don’t it’s very frustrating. I work half days on Saturdays and they don’t open when I’m free on Sundays. No time.” (HChol) |
| **Can spend money on other things** | “I don’t have money to see a doctor. To find the money, I’ll have to cut down on my drinking. I drink 4 to 5 days a week and 4 to 5 bottles every day. Sometimes I even reach 8. It’s expensive but no choice, can’t stop. So I don’t have money to see doctor, how to go?” (HTN) |
| **Attitudes** | |
| *Barriers* | |
| **Fatalism** | “If get these diseases, nothing can be done anyway. You don’t know when you get it, so you can’t do anything anyway. So I think that no need to check, when you’ve got to go you’ve got to go.” (DM) |
| **Fear of diagnosis and/or treatment** | “Yes, I'm worried because if you have diabetes, your skin will rot. If you were to be diagnosed with diabetes your life would be horrible! You can't eat any sweet stuff at all and if you have a wound, you'll be worried the wound doesn't heal and the skin rots. I’m very scared of diabetes, more than thyroid and cancer, because with diabetes, if you cut your hands or legs you might lose the limb.” (DM) |
| **Too old to go for screening** | “We are old okay? So what’s the point of going? If know, so what? Only a few more years anyway.” (HChol) |
| **Traditional medicine is better** | “No. I haven’t checked for anything at all. If you have high blood pressure, you take a slice of *assam,* ^a^ put it in the water, drink it and you will be good. I see the *sinseh*, ^b^ he’s good with the herbs and medications. If you have abdominal pain, you should take ginger slices. If high blood pressure, *assam* ^a^ water. My friends were all cured. If you are scared of hypertension, must drink the *assam* ^a^ water every day.” (HTN) |

HTN: Quotations from patients in reference to hypertension screening using sphygomanometers

DM: Quotations from patients in reference to diabetes screening using fasting blood glucose test

HChol: Quotations from patients in reference to dyslipidemia screening using fasting blood lipid test

^a^ *Assam*: peel of the tamarind fruit, used in traditional medicine in Southeast Asia

^b^ *Sinseh*: traditional Chinese medicine practitioner
